# Monitoring for the genetic structure of Mezen breed of horses in terms of DNA microsatellites

**DOI:** 10.18699/VJ21.024

**Published:** 2021-03

**Authors:** N.V. Vdovina, I.B. Yuryeva

**Affiliations:** N. Laverov Federal Center for Integrated Arctic Research of the Ural Branch of the Russian Academy of Sciences, Arkhangelsk, Russia; N. Laverov Federal Center for Integrated Arctic Research of the Ural Branch of the Russian Academy of Sciences, Arkhangelsk, Russia

**Keywords:** Mezenskaya breed of horses, monitoring, genetic diversity, microsatellite DNA, allele, genotype, мезенская порода лошадей, мониторинг, генетическое разнообразие, микросателлиты ДНК, аллелофонд, генотип

## Abstract

Mezenskaya horse (Mezenka) is Russia’s aboriginal breed. It is a domestic selection in the northern territories of Arkhangelsk region. The breed is perfectly adapted to the conditions of the Far North, and has a number of valuable economic and biological qualities. At present, it has a limited gene pool and is bred only in the Mezensky district, where one gene pool-breeding farm is operating and so is a number of basic farms, where selection and breeding activities take place with the breed. Due to a small population of Mezen horses, the challenge of preserving its intra-breed diversity is very urgent. To determine the degree of genetic variability in the Mezen population, the alleles-fond was monitored. A comparative analysis of the genetic structure of the breed was done on DNA microsatellites at time-intervals of 10 years (2000, 2010 and 2020). Crista samples of 198 horses were studied in specialized laboratories. It was established that the breed has wide genetic diversity in 17 loci of nuclear DNA. The population’s alleles-fond includes from 128, 139, and 133 alleles respectively (with an average value of 7.53, 8.18, and 7.82 alleles per locus). The most common alleles are AHT4O, AHT5N, ASB2K, ASB23S, CA425N, HMS1J, HMS1M, HMS2H, HMS3M, HMS7L, HTG4M, HTG6O, HTG7K, HTG7O and LEX3M. Mezen horses revealed 6 rare, lowfrequency (0.004–0.056) alleles not found in the horse populations of domestic selection. The average value of the polymorphic level (Ae) in the breed over the years is 4.16, 4.21 and 4.06, respectively. The highest polymorphism is found in locus ASB17 (6.49–6.90–6.76); the lowest, in locus HTG6 (1.71–1.66–1.67) and HMS7 (1.77–1.95–1.77). A slight deficit of heterozygous genotypes (Fis = 0.003) was observed in Mezen horses in 2010. In 2000 and 2020, the observed heterozygosity (Ho) exceeds the expected value (He), which indicates the absence of intra-population inbreeding (Fis = –0.014 and –0.011, respectively). The results obtained testify to the effectiveness of breeding activities carried out to preserve, improve and maintain genetic diversity in the population.

## Abstract (ru)

Мезенская лошадь (мезенка) – аборигенная порода России. Была выведена методом народной селекции в северных районах Архангельской области. Порода прекрасно приспособлена к условиям Крайнего Севера и обладает рядом ценных хозяйственно-биологических качеств. В настоящее время имеет ограниченный генофонд и разводится в чистоте только в Мезенском районе, где функционируют одна генофондно-племенная ферма и ряд базовых хозяйств, в которых осуществляется селекционно-племенная работа с породой. В связи с малочисленностью популяции мезенских лошадей проблема сохранения ее внутрипородного разнообразия очень актуальна. Для определения уровня генетической изменчивости в породе проведен мониторинг ее аллелофонда. Сравнительный анализ генетической структуры породы выполнен по микросателлитам ДНК с периодичностью в 10 лет (2000, 2010 и 2020 гг.). В специализированных лабораториях были исследованы образцы волос 198 лошадей. Установлено, что порода обладает широким генетическим разнообразием по 17 локусам ядерной ДНК. В исследуемые годы аллелофонд популяции включал 128, 139 и 133 аллеля соответственно (при среднем значении 7.53, 8.18 и 7.82 аллеля на локус). Наиболее распространенными в породе являются аллели AHT4О, AHT5N, ASB2K, ASB23S, CA425N, HMS1J, HMS1М, HMS2Н, HMS3М, HMS7L, HTG4М, HTG6О, HTG7K, HTG7О, LEX3М. С малой частотой (0.004–0.056) у мезенок было обнаружено 6 редких аллелей, которые не выявлены в популяциях лошадей отечественной селекции. Среднее значение уровня полиморфности (Ае) в породе по годам составляет 4.16, 4.21 и 4.06 соответственно. Наивысший его показатель приходится на локус ASB17 (6.49–6.90–6.76), наименьший – на локусы HTG6 (1.71–1.66–1.67) и HMS7 (1.77–1.95–1.77). Незначительный дефицит гетерозиготных генотипов (Fis = 0.003) отмечен у мезенских лошадей в 2010 г. В 2000 и 2020 гг. наблюдаемая гетерозиготность (Но) превышает величину ожидаемой (Не), что указывает на отсутствие внутрипопуляционного инбридинга (Fis = –0.014 и –0.011 соответственно). Полученные результаты свидетельствуют об эффективности проводимых селекционных мероприятий по сохранению, совершенствованию и поддержанию генетического разнообразия в популяции.

## Introduction

Presently, considerable attention is paid to the preservation of biological diversity, as the “creative effort” of humans has brought many animal species onto the brink of extinction. Populations of local breeds that bear in their genome valuable qualities adapting them to the conditions of the area where they had been developed have reduced significantly. The main cause for the decline of populations and extinction of aboriginal breeds is their inability to compete with modern farm breeds and global breeds in terms of productivity (Fewson, 1979; Simon, Schulte-Coerne, 1979; Lehane Leigh, 1981; Avon Laurent, 1983; Minchev, Dzhurbineva, 1983). The depletion of genetic resources leads to dramatic changes in the gene pool and, above all, to reduction of genetic variability (Altukhov, 2004; Moiseeva et al., 2006; Gendzhieva, Sulimova, 2009; Stolpovskiy, Zakharov-Gezekhus, 2017).

Investigation of genetic characteristics of several local horse breeds in Russia demonstrates that at the present stage of their development these breeds have high levels of genetic diversity and allele pools characteristic of the breeds. For instance, 145 alleles for 17 microsatellite DNA loci were identified in the genotypes of horses of the Yakut breed, that is, 8.53 alleles per locus on the average (Kalinkova et al., 2015). The population of Kyrgyz horses has a vast set of alleles, 135 (Isakova et al., 2018). In Bashkir horses, 130 alleles, or 9.29 alleles per locus, were identified in 14 short tandem repeat (STR) loci (Kalinkova et al., 2016). The population of TransBaikal horses has high genetic diversity indices. With 116 alleles in 14 satellite DNA loci, the level of polymorphism (Ae) of the breed amounts to 5.29, and the observed heterozygosity (Ho) amounts to 0.786 (Kalashnikov et al., 2017a). Acharacteristic feature of aboriginal horse breeds is that their genotypes bear rare and unique alleles not found in farm breeds. Unique alleles were identified in the Buryat, Khakassian (Kalashnikov et al., 2010), Trans-Baikal (Kalashnikov et al., 2017a), Altai, Bashkir, Yakut (Khrabrova, 2015), and Tuvan (Chysima et al., 2017) horse breeds.

The Mezen horse (Mezenka) is one of the local Russian breeds. The area of its origin and present distribution is the Mezensky district, situated in the northeast of Arkhangelsk Oblast. The breed was developed by local inhabitants, and it was perfectly adapted to the harsh conditions of the Far North during its historical formation. The Mezen horse is easy to keep, feed, and manage. It shows good disease resistance, retains its nutritional status in winter, has universal working abilities, and can walk through deep snow and sticky clayey soil. In the 17–19 centuries, Mezen horses were widespread in the Arkhangelsk region. The mechanization of agriculture and termination of the state support of horse breeding in the second half of the 20th century led to a decline in the populations of native horse breeds in Russia, including the Mezen horse breed. By the early 1990s, the breed was preserved only in the Mezensky district. 

At present, the population of Mezen horses has a limited gene pool; it is an intrabreeding population of small size (187 mares as of 01.01.2020). According to the classification of breeds by the degree of risk presented in the Food and Agriculture Organization of the United Nations (FAO) report of 2015, it is included in the “critical status” category (with less than 200 female animals) (FAO, 2015).

Activities on the restoration and preservation of the genetic diversity of the Mezen horse breed have been conducted since 1993. A specialized breeding farm has been operating in the region since 1994, and its main aim is to preserve the intrabreed diversity of the population. The stallions and the mares at the farm include representatives of the breed from various communities of the Mezensky district, characterized by a certain genetic pattern. Important stages of breeding are the exchange of breeding material among the farms raising Mezen horses and the identification of new genetic resources of the breed. The assessment of the genetic situation in the population conducted earlier on the base of polymorphic proteins and blood types revealed the presence of considerable intrabreed diversity (Khrabrova et al., 2005; Yuryeva et al., 2005). However, over the past twenty years, the number of farms and horses in the Mezensky district decreased significantly, and therefore breeding activities engage a small number of stallions and mares. With the increasing likelihood of inbreeding, it may lead to the loss of individual genes and decrease in genetic variability in the breed.

The aim of this study was to monitor the genetic structure of the Mezen horse breed by microsatellite DNA loci and to assess the genetic diversity of the population.

## Materials and methods

The material for the study comprised genetic certificates with test results for 17 microsatellite DNA loci from Mezen breed horses. Only data for the animals included in the breed at the beginning of 2000 (n = 62), 2010 (n = 163), and 2020 (n = 143) were processed. DNA samples obtained from the biological material of horses were genotyped at the Laboratory of Genetics of the All-Russia Research Institute of Horse Breeding and at the Molecular Certification Laboratory of the Gordiz company in 2007–2019. DNA was isolated from hair follicles with Diatom™ DNA Prep, ExtraGene™ DNA Prep (both from Isogen Laboratory, Moscow), and COrDIS SPRINT kits (Gordiz, Moscow).

The samples were analyzed by PCR with multiplex kits for genotyping horses from the Stock Marks and COrDIS Reindeer companies for 17 microsatellite loci: VHL20, HTG4, AHT4, HMS7, HTG6, AHT5, HMS6, ASB23, ASB2, HTG10, HTG7, HMS3, HMS2, ASB17, LEX3, HMS1, and CA425 (van de Goor et al., 2010). PCR was carried out in a 2720 Thermal Cycler. The amplificates were resolved by capillary electrophoresis in an ABI 3130 automatic genetic analyzer (Applied Biosystems). The results were identified using a standard DNA profile and data from international comparison tests (Horse Comparison Tests) (van de Goor et al., 2010).

The genetic analysis of the population was performed as in (Khrabrova et al., 2011). The following indicators were calculated: frequencies of alleles and genotypes, polymorphism level (Ae), expected (He) and observed (Ho) heterozygosity levels, and fixation index (Fis). Statistical analysis was conducted on a PENTIUM-MMX-166 PC with Excel 7.0 software.

## Results and discussion

The time variation of the Mezen horse genetic structure traced by 17 microsatellite DNA loci demonstrates its broad allelic diversity. In 2000, 128 alleles were identified in the Mezen horses. The horses included in this research were born in six communities of the Mezen district, and they had a certain set of alleles in their genotypes. A more than twofold increase in the number of examined horses and expansion of their range of origin to 11 communities permitted us to identify 139 alleles in 2010. The new alleles were identified with frequencies of occurrence from 0.003 to 0.031. In 2020, 133 alleles were identified in the examined horses. The number of alleles decreased over the past decade due to the disappearance of rare (p < 0.05) variants from the population: AHT4L and AHT4N, ASB23Q and ASB23R, CA425O, HMS2Y, HMS3N, and HTG6P. At the same time, in 2020, two new alleles (HTG6G and HTG10T ) were discovered; they were absent from the horses examined in the first two rounds of the study. The alleles identified in the Mezen breed are shown in Table 1

**Table 1.**
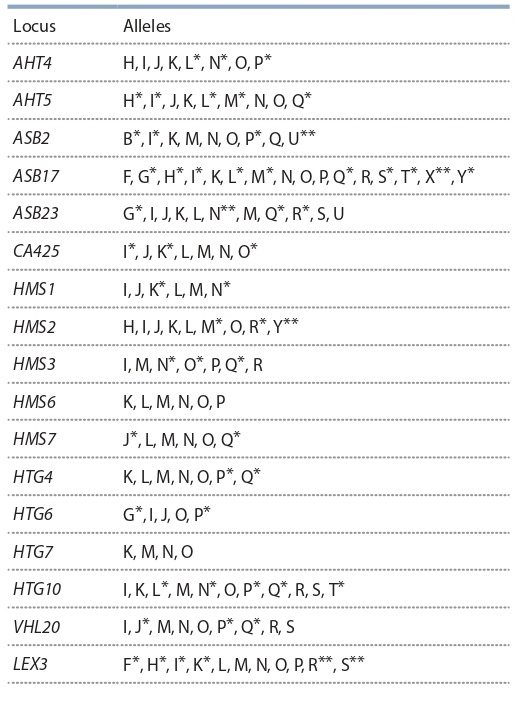
Alleles identified in Mezen breed horses (n = 165) * Alleles rare in the breed: frequencies below 0.05. ** Unique alleles.

As for the loci, the minimum number of alleles was detected at HTG6 and HTG7 (4 alleles in each throughout the years of the study), the maximum number was at the ASB17 locus (13 alleles in 2000 and 16 alleles in each of 2010 and 2020). The numbers of identified alleles per locus averaged over each year were 7.53, 8.18, and 7.82, respectively.

The commonest alleles in the breed are AHT4O, AHT5N, ASB2K, ASB23S, CA425N, HMS1J, HMS1M, HMS2H, HMS3M, HMS3R, HTG4M, HTG7K, HTG7O, LEX3M, and VHL20N. The frequencies of their occurrence range from 0.258 to 0.569. More than 70 % in the structure of their loci is made up by the HMS7L and HTG6O alleles. The frequencies of rare alleles in the population vary from 0.003 to 0.048. Low frequencies (0.003–0.041) are characteristic of six unique alleles found in the Mezen genotype that had not been detected in other domestic horse breeds (van de Goor et al., 2010). In all the analyzed years, the allele pool of Mezen horses contained unique alleles ASB17X and LEX3S. In 2000 and 2010, the HMS2Y allele was detected, and in 2010 and 2020, alleles ASB2U, ASB23N, and LEX3R

Comparative analysis of the genetic structure of Mezen horses over the time span of the study revealed significant ( p < 0.001) differences in the frequency of occurrence of individual allelic variants. New alleles appeared at several loci; as a result, allele frequencies increased or decreased. In particular, alleles AHT5J, ASB17K, CA425I, CA425M, HMS1L, HMS6K, and HTG10O, identified in 2000 at frequencies 0.121–0.213, were 1.2 times less frequent in the population in 2010 and 1.5 to 2.2 times less frequent in 2020. Inversely, alleles AHT5K, ASB2M, ASB17R, ASB23I, CA425L, CA425N, HMS1J, HMS3M, and HTG10I at the second and the third steps of the study occurred at frequencies higher than at the first step by factors 1.1–1.2 and 1.3–1.5, respectively. Over twenty years, the frequencies of the typical HTG7O (38.0 to 49.0 %) and HMS3M (27.5 to 38.7 %) alleles increased significantly, while the frequencies of HTG7K and LEX3M decreased by 9.6 and 13.6 %, respectively.

A significant difference (p < 0.05) between the examined groups was also noted in the number of genotypes (allelic variants). In 2000, 278 variants were tested at 17 microsatellite DNA loci in the Mezen horses, the numbers of which in the loci varied from 6 (HTG6 ) to 30 (ASB17 ). By 2010, the number of genotypes increased to 387. The number of genotypes identified in 2020 was 345. At the same time, the analyzed population lacked 44 variants present in the horses examined in 2000, but 111 new ones were discovered. The most significant increase over the past 20 years was noted in loci HTG10 (from 20 to 29), ASB17 (from 30 to 46), and LEX3 (from 8 to 27).

The conducted genetic analysis of the population demonstrated that due to the wide genetic diversity in the population of the Mezen horses the level of polymorphism, characterizing the number of effective alleles, remained high throughout the study (Table 2).

**Table 2.**
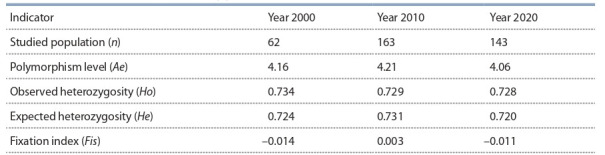
. Genetic and population characteristics of the Mezen breed of horses in terms of DNA microsatellites, 17 loci, by years

The maximum number of effective alleles (Ae) over years was observed in the highly polymorphic locus ASB17 (6.49, 6.90, and 6.76), in which homozygous genotypes constituted 11.3 to 16.7 %. In loci HTG6 and HMS7, alleles O (0.742–0.754) and L (0.697–0.738), respectively, were predominant, thus accounting for their low polymorphism (1.66–1.95). Since the homozygous genotypes HTG6OO and HMS7LL dominate in these loci with frequencies above 50 %, their levels of observed heterozygosity (Ho) were low, 37.4 to 47.5 %, respectively. In other loci, the levels of polymorphism in 2000 varied from 2.53 (LEX3) to 5.43 (HMS6 ); in 2010, from 2.96 (HTG7) to 5.29 (HMS6 ); and in 2020, from 2.74 (HTG7 ) to 5.49 (HMS2); the observed heterozygosity varying from 57.1 to 88.7 %.

Generally, heterozygous genotypes prevailed in the population in 2000. This was proven by the value of observed heterozygosity (0.734), which was higher than the predicted level (0.724), and the negative value of the fixation index (Fis = –0.014). This indicator demonstrated the presence of genetic balance in the breed and the absence of intrapopulation inbreeding. Aslightly reduced value of heterozygotes (Fis = 0.003) was observed in the Mezen horses in 2010. In this study, the actual heterozygosity at loci ASB23 and HTG7 corresponded to the predicted value, and at several loci (HTG4, HMS7, HTG6, AHT5, ASB2, HMS3, and ASB17 ), the predicted heterozygosity was higher than the observed one. By 2020, the genetic balance in the population was restored. This was confirmed by the negative values of fixation index at most loci and on the average over the breed (Fis = –0.011). The predominance of heterozygous genotypes proved the effectiveness of the breeding activities aimed at the preservation and maintenance of genetic diversity in the breed. 

Molecular tracing of the time variation of the Mezen horse allele pool at DNA microsatellite loci shows that the breed, like other local horse breeds, has a high level of allelic diversity in most of the loci tested and a wide genetic variability. The population has its specific genetic profile, which differs from some other local breeds (the Altai, Bashkir, Buryat, Vyatka, Trans-Baikal, Pechora, Tuvan, Khakassian, and Yakut horse breeds) (Khrabrova et al., 2009; Kalashnikov et al., 2010; Khrabrova, 2016; Blokhina et al., 2018; Yuryeva et al., 2018). Thus, the genetic structure of the Mezen horse breed does not include the AHT4L, AHT4P, ASB17Q, HMS7K, or HTG6G alleles, which are found in the genotypes of the Trans-Baikal (Kalashnikov et al., 2017a), Kalmyk (Kalashnikov et al., 2017b), Yakut (Kalinkova et al., 2015), and Bashkir (Kalinkova et al., 2016) horse breeds. The AHT5M, HTG7M, and HTG10L alleles ( p < 0.05), rare in the Mezen horse breed, are characteristic of the mentioned populations. Conversely, the CA425L allele, widespread in the Mezen breed (frequency 0.214), was designated as rare in the Yakut horse breed and was not detected in Bashkir horses. The ASB23Q and HTG10T alleles, which are present at low frequencies in the Mezen population, are observed only in the genetic structure of Bashkir horses, and the ASB17Y allele occurs in the Yakut horse breed. The AHT5H, ASB17X, HMS2Y, HMS6J, LEX3R, and LEX3S alleles were detected only in the Mezen horse breed.

At present, agricultural enterprises of the Mezensky district have stallions and mares with rare allelic variants of microsatellite DNA. Some of them have two to five rare alleles in their genotypes. The replication of these alleles through their carriers and identification of new genetic resources in the region will allow not only the preservation but also the expansion of genetic diversity in the small population of the Mezen horse breed.

## Conclusion

Monitoring of the genetic structure of the Mezen horse breed revealed certain changes in the numbers of alleles and their combinations in the allele pool, as well as in the frequencies of their occurrence. The breed has a high level of allele variability and a certain genetic profile for DNA microsatellites, which is an important factor in maintaining the gene pool in a small population.

## Conflict of interest

The authors declare no conflict of interest.
